# Global health economics: The Equitable Impact Sensitive Tool (EQUIST) – development, validation, implementation and evaluation of impact (2011 to 2022)

**DOI:** 10.7189/jogh.13.04183

**Published:** 2023-12-15

**Authors:** Mickey Chopra, Lakshmi N Balaji, Harry Campbell, Igor Rudan

**Affiliations:** 1 The World Bank, Washington, District of Columbia, USA; 2 United Nations Children Fund (UNICEF), New York, New York, USA; 3 Centre for Global Health, Usher Institute, University of Edinburgh, Edinburgh, UK

## Abstract

**Background:**

The Equitable Impact Sensitive Tool (EQUIST) was developed to address the limitations of the traditional cost-effectiveness analysis (CEA) in global health, which often overlooked equity considerations. Its primary aim was to create more effective and efficient health systems by explicitly incorporating equity as a key driver in health policy decisions. This was done in response to the recognition that, while CEA helped reduce mortality rates through interventions like childhood vaccinations, it was insufficient in addressing growing inequalities in health, especially in low- and middle-income countries (LMICs).

**Methods:**

The development of EQUIST involved a multi-stage process which began in 2011 with the recognition of the need for a more nuanced approach than CEA alone. This led to a proposal for creating a tool that balanced cost-effectiveness with equity. The conceptual framework, developed between March and May 2012, included assessments of intervention efficiency by equity strata, effectiveness, impact, and cost-effectiveness. Key to EQUIST’s development was its integration with other data science platforms, notably the Lives Saved Tool and the Marginal Budgeting for Bottlenecks tool, allowing EQUIST to draw on comprehensive data sets and thus enabling a more detailed analysis of health interventions' impacts across different socio-economic strata.

**Results:**

EQUIST was validated in 2012 through applications in five representative countries, demonstrating its ability to identify more equitable and cost-effective health interventions which targeted vulnerable populations, leading to more lives saved compared to traditional methods. It was then used to develop investment cases for the Global Financing Facility, resulting in significant funding being made available for maternal and child health programmes. Consequently, EQUIST directly influenced the development of national health policies and resource allocations in over 26 African countries.

**Conclusions:**

EQUIST has proven to be a valuable tool in developing health policies that are both cost-effective and equitable. In the future, it will be further integrated with other tools and expanded in scope to address broader health issues, including adolescent health and human immunodeficiency virus/acquired immunodeficiency syndrome programme planning. Overall, EQUIST represents a paradigm shift in global health economics, emphasising the importance of equity alongside cost-effectiveness in health policy decisions. Its development and implementation have had a tangible impact on health outcomes, particularly in LMICs, where it has been instrumental in reducing maternal and child mortality while addressing health inequities.

This paper summarises the development, validation, implementation, and evaluation of the Equitable Impact Sensitive Tool (EQUIST), a health policy decision tool that explicitly considers equity as a main driver of developing more effective and efficient health systems. It represents an important advance in global health economics over the past decade.

The landmark publication “Investing in Health” from the World Bank in 1993 [1] was influential in exposing the interconnections between health and wealth, while strongly promoting the central importance of efficiency in prioritisation of health interventions. This was captured by the development and using cost-effectiveness analysis (CEA) as a guiding principle of investing in population health and prioritising health interventions. This situation then remained largely unchanged for nearly two decades.

The widespread use of CEA further focused attention on highly cost-effective interventions such a childhood vaccination and thus contributed to further reductions in mortality rates. However, by the early 2000s, it became clear that progress was decelerating and– of even greater concern – inequalities were widening. Despite global efforts to reduce maternal, neonatal, infant, and child mortality rates within the United Nations (UN) Millennium Development Goal 4, they remained unacceptably high in many low- and middle-income countries (LMICs) [2]. A major challenge for agencies like the United Nations Children’s Fund (UNICEF) was to plan and deliver maternal and child health interventions that are both cost-effective and equitable.

Equity, i.e., the distribution of resources based on the needs of the recipients, is a key concept in reducing health inequalities. While interventions are more complicated and costlier to deliver to poor rural communities, health needs – and therefore potential impacts of interventions in these communities – are typically greater, meaning a given level of intervention would make a more marked difference. This results in a highly complex balance between health needs, measured through potential impact of health interventions, and the cost of intervention delivery and effectiveness in reducing maternal and child mortality. In 2010, research conducted at UNICEF showed that the simple CEA approach may no longer be sufficient to address these complexities and enable intervention prioritisation and health policy and planning in low resource settings [3].

## YEAR 2011

### The need for the EQUIST and the first investment in its development

In 2011, three authors of this paper (IR, HC, and MC) began working on a Lancet series on childhood pneumonia and diarrhoea together with researchers from UNICEF and other agencies. This work aimed to identify the overall global, regional, and national burden and the key interventions that are cost-effective against childhood pneumonia [4,5], but also went on to highlight key barriers to the reduction of inequities in access to health services between the most deprived and wealthier groups in society [6].

The study was carried out within broader efforts to reduce global child mortality. Working as the Chief of Health at UNICEF, one of the authors (MC) realised that there may be shortcomings at the core of CEA. He published a paper with several co-authors based at UNICEF in late 2010 [7] showing that, while reducing child mortality, some countries were increasing inequity among children, while others were reducing inequity. This surprised the global health research community, because it was generally thought that reducing child mortality would necessarily also reduce inequity among children. Therefore, a need to modify the approach based on CEA alone arose through evaluation of successes in child mortality reduction globally.

This led to a question that was difficult to answer for anyone committed to global health: if there was, say, US$1 million available for investing in a health intervention, and if it could reduce 100 deaths among the well-off children in the capital city of a country, or 90 deaths among the poorest and most underprivileged children in remote areas, what would be the appropriate investment decision?

The mathematics behind this is simple: it is more cost-effective to save 100 lives for the same amount of money, regardless of whether the saved children are affluent or not. This is because the efficiency and quality of delivery of health interventions in the affluent part of the population makes it so – reaching poor children with interventions is costly, and can also be inefficient and ineffective.

The authors of this paper [7] realised that strictly following the principle of CEA in prioritising interventions for child survival may not always align with the need to improve equity among children. Consequently, UNICEF initiated a call for proposals for advanced methodologies to estimate the effects of equity-focused approaches to improve child health, entitled “Development of advanced methodologies and tools to estimate the effects of equity-focused approaches to improve child health”.

This competitive call for funding awarded one of the authors’ (IR) proposal entitled “Developing a user-friendly Excel-based program to model the complex interplay between the cost of intervention scale-up, the targeted population by equity strata, and the burden of disease averted”. The project’s goal was to develop a tool that could consider both cost-effectiveness and equity when prioritising child health intervention.

## YEAR 2012

### The development of the EQUIST

Between March and May 2012, two authors (IR and MC) developed the conceptual framework that could address this problem, while another (HC) assisted in further improving the framework. This resulted in the first related joint publication, entitled “Understanding the determinants of the complex interplay between cost-effectiveness and equitable impact in maternal and child mortality reduction” [8].

**Figure 1:**
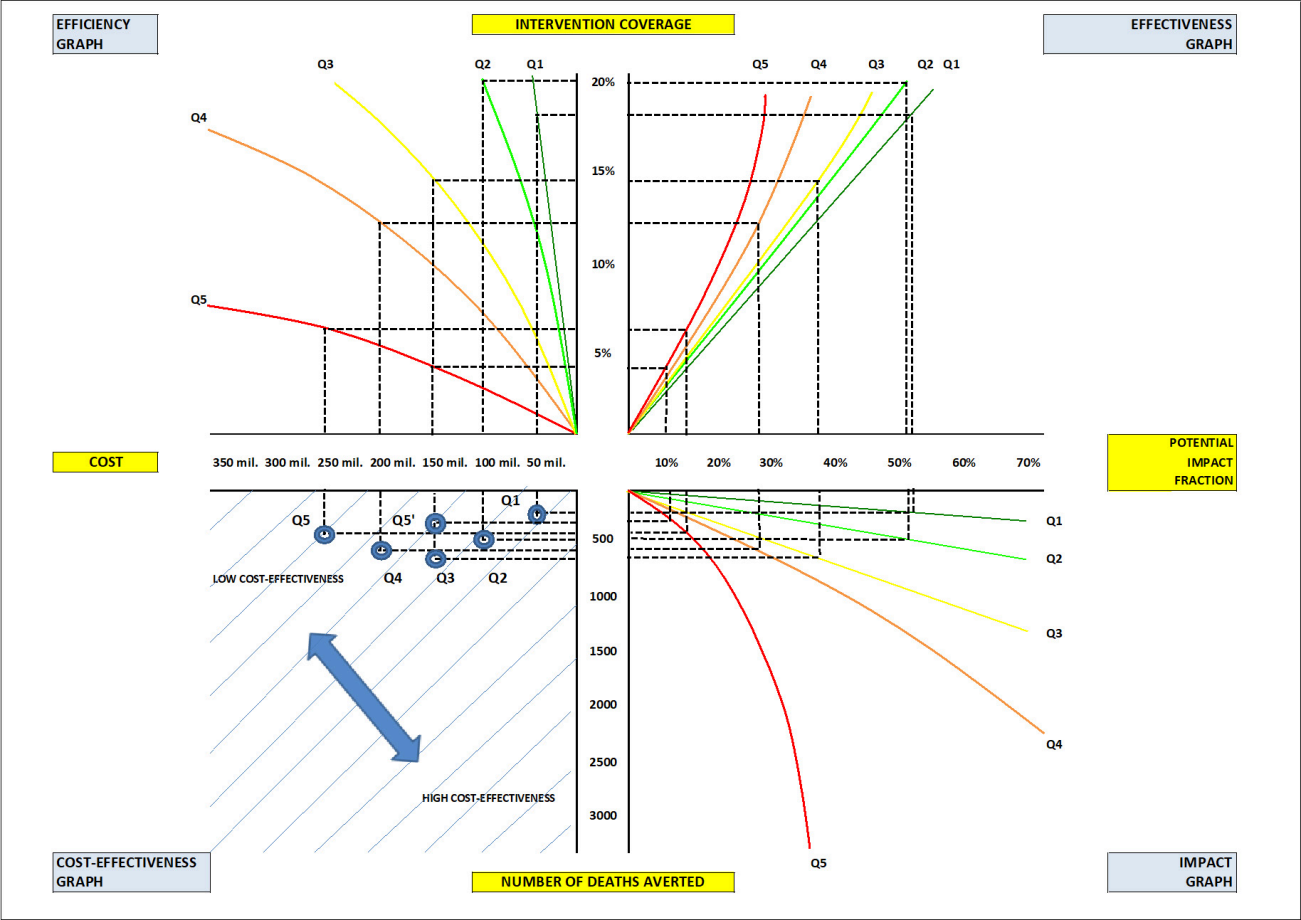
The EQUIST approach to prioritising the effectiveness of interventions. The detailed explanation of the framework is given elsewhere [8], as is an example of its use in practice [9].

The aim of the new framework (**Figure 1**) was to add the criterion of the impact on equity to the criterion of cost-effectiveness when prioritising investments in health interventions. The result of this framework was EQUIST – a health economics tool that allows comparative examination of strategies that are both cost-effective and equitable [8]. It assumes that equity-focused strategies that remove quantifiable bottlenecks can lead to improvements in the coverage of high-impact interventions.

EQUIST’s core algorithm (**Figure 1**) takes the decision-making process through the assessments of efficiency of intervention delivery by equity strata, effectiveness, impact, and cost-effectiveness. Its output included determination of both cost-effectiveness and the impact on equity among the competing interventions, allowing for those that can equitably reach vulnerable populations within available resources to be identified and promoted [8].

Thus, the paper by Chopra et al. [8] presented the conceptual framework for the EQUIST, which models the complex interplay between three key determinants of a health intervention discussed above: efficiency, clinical effectiveness, and impact. These determinants predict the cost-effectiveness of an intervention across different socio-economic strata in the population, and thus allowing the tool to identify key bottlenecks that constrain the coverage of interventions and target the most cost-effective and equity-focused strategies that can address them. In this way, EQUIST provides critical data for national and international policymakers for planning equitable and cost-effective interventions to reduce maternal and child mortality, particularly among more deprived populations.

At the time when EQUIST was being developed, there were no agreed-upon approaches to prioritising across the spectrum of proven, cost-effective health and development programmes at the national or local level in contexts where immediate needs far exceed the level of funding and resources available. Operational teams require a more detailed framework and guidance for systematically comparing and evaluating investment options across the health and development spectrum, while taking specific contextual factors into consideration.

### Validation of the EQUIST

The tool was then validated from August to October 2012 by applying it simultaneously in five countries representative of larger World Health Organization (WHO) regions: Nigeria (sub-Saharan Africa region), Egypt (Eastern Mediterranean region), Bangladesh (South-East Asian region), Cambodia (Western Pacific region), and Peru (Americas region) [9]. These countries were selected because of their large size and relatively adequate information reported by equity strata. Three authors (IR, MC, and HC) decided to focus the validation on a single disease – pneumonia, which was still the leading cause of child deaths globally. To allow appropriate scrutiny, a single intervention was studied, namely integrated community case management. The entire record of validation of the EQUIST tool was documented and published in our second related joint publication [9].

This research clearly demonstrated that the “mainstream” approach of targeting easier-to-reach parts of society was never the most cost-effective method for achieving impact. For example, in Peru, a US$1 million investment would lead to 5209 lives saved using a “mainstream” model targeting the wealthiest quintile of society, while the same investment would save 7191 lives using an equity-promoting model targeted at the poorest quintile [9].

This paper also demonstrated how to conduct an EQUIST scenario analysis that could help identify cost-effective and equitable intervention packages for any single disease, along with exposing the bottlenecks in cost, efficiency, and effectiveness, and strategies to address them. The authors then proposed to develop a flexible approach that would allow country-level teams to derive the optimal mix based on their local information and knowledge. It was necessary to develop an algorithmic process to assist country teams to objectively select the optimal mixes of interventions for different epidemiological profiles and for the different delivery platforms (such as community health workers, outreach, primary health care, etc.) based upon EQUIST analysis.

### Digitalisation of the EQUIST

To this end, the next step of EQUIST tool’s development was to automate entry of relevant data into an integrated data science platform. This built upon other analytic work, especially that of WHO's and UNICEF's Child Health Epidemiology Reference Group (CHERG) that was developing estimates of the burden of global, regional, and national child health morbidity and mortality [2,4,10,11] and intervention effectiveness [12-14]. Their estimates populated the Lives Saved Tool (LiST), that was being developed at the Johns Hopkins University at the time [15,16].

Between 2009 and 2012, two authors (IR and HC) held a grant from the Bill and Melinda Gates Foundation entitled “Analysis of landscape and potential impact of diagnostics for neonatal infections”, from which they allocated a sub-grant to Johns Hopkins University so as to add the cost-effectiveness and equity modules to the LiST. This newly-equipped tool was eventually used to populate EQUIST with the key information on global child health indicators.

## YEAR 2013

### Integration between the EQUIST and the MBB tool

In 2013, the data science platform was being developed at the UNICEF’s Innocenti Centre. The key challenge was to populate EQUIST with the detailed information that it required, which included the integration with existing data science platforms that contained “big data” to inform health policy globally. The first proper integration was done between the EQUIST platform and the Marginal Budgeting for Bottlenecks (MBB) tool [17], an analytical costing and budgeting tool developed in 2002. MBB helps countries develop their health plans by considering the most effective interventions, cost, and budget marginal allocations of their implementation to health services and assess their potential impact on health coverage, health-related Millennium Development Goals, and health outcomes of the poor [18]. It builds on the high impact interventions to reduce mortality developed in several studies, including The Lancet series on Child, maternal and neonatal survival [2].

The MBB tool has been developed in the context of Highly Indebted Poor Country Initiative and Poverty Reduction Strategy Papers, and mainly addresses the following six questions:

1. Who does what? Which high-impact interventions can be integrated into existing providers/service delivery arrangements to accelerate progress toward the health Millenium Development Goals?
2. Equity?
3. What are the major hurdles or “bottlenecks” hampering the delivery of health services, and what is the potential for their improvement?
4. How much money is needed for the expected results?
5. How much can be achieved in health outcomes such as mortality reduction by removing the bottlenecks?
6. Which amounts of financing is it possible to mobilise, and how should these be allocated and channelled?

The MBB tool builds on the theoretical knowledge of the Tanahashi model of evaluating health service delivery performance based on five determinants of the health system [19], the implementation of effective interventions, also called high impact interventions [20], and fiscal space, where governments can collect the money to implement the marginal cost required.

The MBB tool was initially developed by teams from the World Bank's Africa region, the South Asia region, and the Health Nutrition and Population Anchor, in collaboration with UNICEF and the WHO. It is a health systems performance monitoring tool which had been in use in the West African Region since the 1990s. Eventually, WHO began developing their own approach, while the MBB was upgraded with the option to use the LiST tool to calculate impacts from 2008 [15,16]. Since then, discussions on producing an integrated model that would re-unify different models proposed by different agencies (UNICEF, WHO, Joint United Nations Programme on HIV/AIDS (UNAIDS), United Nations Population Fund (UNFPA)) into one comprehensive planning and budgeting tool have re-intensified, while the MBB tool was made available in three languages. The MBB tool runs on MS Excel, while DevInfo [21], an online support platform where the latest version could be downloaded along with on-the-job support (developed by another author of this paper (LB)), was tasked to provide support to users.

The MBB tool has been used at national and sub-national levels in more than 17 countries across Africa and Asia to prepare mid-term expenditures frameworks, investment cases, child survival strategic plans, and/or national health plans. It consists of five key steps:

1. An assessment of the key indicators of your health system at the baseline;
2. Identification of system-wide supply and demand bottlenecks;
3. Selection of interventions and the estimation of the expected impact of each intervention on survival rates;
4. Selection of the types, quantities, and costs of additional inputs;
5. Analysis of budgetary implications and the comparison of marginal costs to the “fiscal space”.

The integration of the MBB tool into the EQUIST online data science platform has addressed many needs required for the latter to become operational and provide health policy advice in different low-resource contexts. However, aside from the health systems element and the context definition, it still required considerable technical input on the wide range of interventions and their effectiveness and efficiency, as well as detailed data on the burden of disease and disability. For this, collaboration with the LiST was needed.

## YEAR 2014

### The communication between the EQUIST and the LiST

By 2014, the MBB tool was embedded within EQUIST and was unavailable for separate use, presenting a major advance supported by both UNICEF and the World Bank. In 2014, LiST was also integrated into the server that supported the EQUIST data science platform.

LiST was an original product/contribution developed by the team from the Johns Hopkins University [15,16]; it was never a part of EQUIST, which simply communicated with LiST and relied on its rich resource of valuable data. LiST was developed as a desktop utility as a part of the Spectrum suite of models and maintained by AvenirHealth, while EQUIST is a web-based utility. An incentive was to make LiST into a web-based tool, which would facilitate the communication between LiST and EQUIST.

Specific points of communication and collaboration that were discussed since EQUIST started communicating with the LiST were, for example, how to build a nutrition module within LiST, so that the impact of nutrition could be assessed; how to develop a model for emergency health needs; and how to move beyond just children and also include the adolescents.

The addition of the MBB and LiST Tools’ capacities, data, and other functionalities and strengths to the EQUIST data science platform created a powerful new device that could be used to assist countries in planning their investments in health interventions at the population level, especially relevant to maternal, newborn, and child health. The next critical step, however, was creating the context in which the EQUIST could demonstrate its usefulness and functionality.

## YEAR 2015

### Creating the context for Implementation of the EQUIST

EQUIST’s large-scale impact on maternal and child mortality reduction became possible after the Global Financing Facility (GFF) was launched in Addis Ababa, Ethiopia, in July 2015. On that occasion, the UN, the World Bank Group, and the Governments of Canada, Norway, and the United States joined country and global health leaders in launching the GFF in support of “Every Woman Every Child” initiative. They announced that “...US$12 billion in domestic, international, private and public funding had already been aligned to country-led five-year investment plans for women’s, children’s and adolescents’ health in the four GFF front-runner countries of the DR Congo, Ethiopia, Kenya and Tanzania” [22].

A press release by the World Bank noted regarding GFF:

“Launched at the Third International Financing for Development Conference, the GFF was a key financing platform in support of the UN Secretary-General’s Global Strategy for Women’s, Children’s and Adolescents’ Health and the UN’s Sustainable Development Goals. At the launch, the World Bank Group announced a new GFF partnership with its International Bank for Reconstruction and Development (IBRD) to raise funds from capital markets for countries with significant funding gaps for reproductive, maternal, newborn, child and adolescent health (RMNCAH). This ground-breaking partnership is expected to mobilize between 3 to 5 USD from the private capital markets for every 1 USD invested into the GFF. The Bill & Melinda Gates Foundation, Canada, Japan, and the United States announced new financing commitments totalling 214 million USD in addition to those previously made by Norway and Canada of 600 million USD and 200 million USD, respectively, to the World Bank Group-managed GFF Trust Fund. Their aim was to help close the 33.3 billion USD annual funding gap for RMNCAH. At the launch, the GFF partners also announced the next group of eight countries – Bangladesh, Cameroon, India, Liberia, Mozambique, Nigeria, Senegal and Uganda – to benefit from the GFF, with the goal of supporting 62 high-burden low- and lower-middle income countries within five years” *[*22*].*

Clearly, the launch of the GFF created a major new financing mechanism for maternal, child, and adolescent health (MNCH). However, to release this major new funding support, each country needed to develop a detailed investment case study (ICS) based on sound data and processes. The GFF and the agencies that launched it broadly considered EQUIST as the most useful and comprehensive tool available to recipient countries to generate successful ICSs. In each new GFF round of funding, the use of EQUIST expanded among the selected countries. Therefore, potential applicant countries were being advised – and their representatives trained at high-level workshops – to use EQUIST in order to maximise their chances of being awarded funding [22].

## YEARS 2016-2018

### Training in EQUIST use and the first country-level contracts

Following the launch of GFF, UNICEF organised intensive capacity-building workshops in EQUIST implementation in 2016-2018. It secured participation from international organisations, national-level offices of the WHO and UNICEF, and high-level policymakers from the countries’ governments. Over the course of these three years, workshops were held in Cameroon, Haiti, Kenya, the Democratic Republic of Congo, Senegal, Mozambique, Guinea-Bissau, Bangladesh, Sudan, Djibouti, Uganda, Lebanon, Malawi, Myanmar, Ghana, and Cote d'Ivoire, enabling them to develop their ICS based on EQUIST for supporting government priorities that will enable their proposals for GFF funding.

Since November 2016, the first among these countries started signing contracts with GFF. They were typically worth tens of millions of US$, although some of them received well over 100 million. The GFF investment cases were paired with International Development Association/International Bank for Reconstruction and Development (IDA/IBRD) loans that together were aimed at reducing child and maternal mortality in these countries. The early examples of EQUIST-based ICSs, which led to the release of substantial funding support for programmes in maternal and child health, are the Democratic Republic of Congo (US$40 million awarded in 2017), Cameroon (US$127 million awarded in 2017), and Mozambique (US$175 million awarded in 2017). However, several other countries also continued to follow their example. There is no doubt that these large investments led to a considerable reduction in maternal and child mortality and morbidity, which was expected to be both cost-effective and equitable. The EQUIST contributed to national debates and discussions on priorities and options, assisting both in terms of suggesting geographic locations for the interventions and strategies for improved coverage and newborn, infant, child, and maternal outcomes. National investment case studies based on EQUIST have estimated the expected lives saved, and they typically ranged from thousands to tens of thousands, depending on the size of the country and the investment made.

### Integration of DHS+ and MICS data into EQUIST

At this point, EQUIST users no longer needed to inspect the Demographic Health Surveys (DHS+) or Multiple Indicator Cluster Surveys (MICS) (the latter originally conceived by one of the authors (LB)) as a primary source of reliable data for their country, as they have also been integrated into the EQUIST data science platform. Country-level capacity building allowed data managers to supplement the pre-populated data with additional reliable information from data sources, such as service provision assessments (SPA), other surveys, and other new, locally available data. Stakeholder discussions based on scenarios developed through the EQUIST allowed local engagement and discussion on bottlenecks in service delivery and barriers to utilisation, and thus the incorporation of local knowledge during the prioritisation exercise.

Simultaneously, EQUIST also retained the functionality of the MBB Tool, allowing it to go through a cascading search and use a proxy in the absence of any indicator, with a clearly defined cascade of those indicators (“supply-related demand”). As an example, oral rehydration use, insecticide-treated bed nets use, or similar indicators were being used as a proxy for a wide range of other missing indicators, to demonstrate the functioning of the health system in the area. By this time, the EQUIST successfully integrated the following rich data sources and components: the original EQUIST algorithm, the MBB tool, the LiST’s data, the DHS+, and the MICS data.

Initially, the development of EQUIST had an impact on UNICEF’s training strategy. After UNICEF expanded the EQUIST model into a user-friendly global data science platform, it subsequently launched training in EQUIST use in Cameroon, Haiti, Kenya, DR Congo, Senegal, Mozambique (in 2016); Guinea-Bissau, Bangladesh, Sudan, Djibouti, Uganda, Lebanon, Malawi, Myanmar (in 2017); and Ghana, Côte d'Ivoire, Chad and Ethiopia (in 2018) [23-25].

The Fourth Global Symposium on Health Systems Research in Vancouver in 2016 [23] and Institutionalizing Community Health Conference Johannesburg in 2017 [24] included sessions where researchers, government officials and international development professionals were trained to use EQUIST. Furthermore, UNICEF developed a core group of trainers in Budapest (2017) and New Delhi (2019) [24].

### A formative assessment of the EQUIST

UNICEF conducted a formative assessment of EQUIST in 2018, which then extended into 2019, principally drawing on the results from an online survey of 88 respondents from 35 countries, particularly those where a field mission was not possible. These countries included Haiti, Bolivia, the Democratic Republic of Congo,, Guinea, Guinea-Bissau, Bangladesh, Sudan, Djibouti, and Uganda, among others. Altogether, 124 individuals in 35 countries, including individuals from UNICEF, UNFPA, USAID, WHO, NGOs, Ministries of Health, and academia, responded to the assessment; many had participated in an EQUIST training. This analysis drew on all survey responded, including those that had received any form of training or orientation on EQUIST.

Key conclusions from the assessment indicated that the tool and approach were relevant to country policies and procedures in health sector planning and priority-setting. Estimations of the number of lives saved by key intervention packages and the related costs have strengthened the health sector planning process in various countries. For countries that have embraced EQUIST, there was a strong likelihood of MNCH outputs and emerging outcomes resulting from the use of the tool. Even though the tool has played a discernible role in strategic planning, prioritisation, and advocacy related to programs for marginalized groups in several countries, it has not always achieved the same visibility as the WHO’s “One Health Tool” and the World Bank/UNICEF’s MBB tool.

It is also important not to equate a tool to an approach. An equity-based approach is still possible, in principle, without using EQUIST. However, EQUIST is a truly useful tool for identifying marginalised populations, and for estimating impact and related costs in terms of lives saved. It can also be used in conjunction with resource mapping of financial information, whereby collected information from development partners regarding pledged or expected financial contributions and technical assistance can enable the health ministry to map funding projections of development partners to programmes and sub-programmes. This critical information would then be used in health budget preparations and submission to the finance ministry.

The assessment identified the following challenges in incorporating EQUIST results into health programming:

1. EQUIST must not be viewed as a “magic bullet” that could solve all problems in planning and implementing strong public health programmes, as it is limited by political commitment, the timing of its use, as well as data quality and availability.

2. Countries and districts need accurate information on the causes of child deaths to generate accurate LiST results; those results can then be used to prioritise interventions and allocate resources. However, the early applications of EQUIST highlighted important gaps in the availability and quality of essential data for program planning and evaluation at the country level. This is particularly true at sub-national level. Moreover, the use of data in policymaking is often challenging. When feasible, data should be collected routinely through nationally representative household surveys. It is also important to maintain consistent indicator definitions across surveys to allow trend analyses. Furthermore, macroeconomic variables such as shipping cost coefficients, distance to hospitals and health facilities, road conditions, and other such logistical data are all important for pinpointing required cost and access to facilities, cost of delivery of commodities, and other factors that impact health system expenditures. As these data are not available in most countries, some (e.g. Cameroon) have employed a multi-sectoral team of experts to efficiently gather all required data in advance of analysis.

3. EQUIST has room for improvement in its reliance on assumptions made for indicators where data are scarce. For instance, due to the missing data, EQUIST’s 2016 Kangaroo Mother Care (KMC) coverage estimates for Cameroon were assumed to be quite high. If the data were not reviewed prior to the final application, the analysis would have been skewed. Consequently, the database was reviewed and additional time was spent to gather the data from a wide range of sources and stakeholders. Another helpful improvement for EQUIST would be the ability to generate multiple different variables in a single graph (e.g. a specific indicator in a region by area of residence (urban vs. rural) and by burden of disease).

4. Data capacity: Intra-country capacity required for using and maintaining EQUIST remains a challenge in many underdeveloped settings, although this capacity is critical for its successful implementation. Inclusive capacity building needs to be ensured for consensus on the outputs. For example, the training in Cameroon took five days, bringing together a range of professionals from the Ministry of Public Health, consultants, and H6 technical partners. They understood the rationale, process, and methodologies used during the prioritisation process. Another useful approach was cross-country knowledge sharing. For example, when Uganda implemented the District Health Information Software 2 (DHIS2), it benefitted significantly from the numerous improvements made during its implementation in Kenya, such as using the trainers from Kenya and the course materials. Positive examples of country collaborations may be applied to the EQUIST’s experience and use. The circulation of practitioners between implementing countries is also a driver of innovation, exposing the established practices to new contexts and ideas.

5. Thematic focus: EQUIST remains limited by its inability to analyse progress and bottlenecks in, for example, adolescent health and young child nutrition. A key justification for broadening EQUIST’s area of focus is that the health sector contributes to only 20% of the population’s health. The remaining 80% is determined by socio-economic factors, health behaviour, physical environment, disaster risk reduction, and climate change. These areas, if integrated with EQUIST, will make the tool stronger and even more applicable.

6. Sustainability: Enhancing further use of EQUIST would require a targeted approach, including the use of key data champions and building a community that would share examples of practical implementation and develop webinars on its use.

## YEAR 2019

By 2019, EQUIST had been used to directly influence national health policy and resource allocation in more than 26 African countries, identifying bottlenecks and developing prioritised strategies to address them. In March 2019, UNICEF jointly trained six governments from sub-Saharan Africa, using the approach where one government representative, one researcher, one member of the local WHO office, and one member of the local UNICEF office comprised a minimum team that participates in this training. They then look at available country-level data on maternal, newborn, child, and adolescent health and use EQUIST to develop ICSs for their countries. The interest to adopt EQUIST was also registered from countries as diverse as Pakistan, Ghana, and Zambia, as its potential implementers.

The main outcomes of interest in LiST were mortality and malnutrition, while adolescent health issues seemed quite small in comparison, necessitating a way to comparatively standardise and measure the issues among adolescents. The WHO moved toward developing analytical tools to build strong equity modelling for the global Accelerated Action for the Health of Adolescents (AAHA) framework. Related to this, the INNOV8 approach to reviewing national health programmes uses the the-step approach to make a health programme more targeted at the most vulnerable. Together with EQUIST’s modelling capability, it was used to develop the Adolescent Health Services Barriers Assessment (AHSBA). Developed jointly by the WHO and UNICEF, AHSBA focuses on disadvantaged adolescents. Country teams were trained in its use in 2019.

### Summer School in Global Health Economics and Priority Setting in Edinburgh

In 2019, The World Bank funded the University of Edinburgh to organise the Summer School in Global Health Economics and Priority Setting, held in Edinburgh from 23 to 27 September 2019. At this school, the experiences with the use of the EQUIST were presented and new ideas for continuing the development and update of the tool were considered. One of the authors of this paper (IR) presented the EQUIST algorithm, the Child Health and Nutrition Research Initiative (CHNRI), and the Planning, Monitoring and Evaluation Tool (PLANET), all of which were investment priority-setting tools developed at the Centre for Global Health at the University of Edinburgh. Some of the invitees who presented on the EQUIST were Dr Shahrouh Sharif, based at the Children’s Scholarship Fund (CSF) in New York, a common point for several capacity-building initiatives in EQUIST, and Dr Jasmit Shah from Aga Khan University Medical College in Nairobi, Kenya, who developed and supported capacity-building activities in Rwanda and Ethiopia. Drs Shariff and Shah had already co-led several training workshops in the use of the EQUIST for African national representatives [25,26].

Other experts joining the Summer School to present their experiences with the EQUIST Tool use were Dr Robert Lucien Jean-Claude Kargougou, a former Minister of Health in Burkina Faso and an EQUIST user; Dr Marie Laurette Agbre-Yace, a paediatrician and expert in EQUIST at Institut National de Santé Publique in Cote d’Ivoire, who was trained in the use of EQUIST and was on the core team that helped put together MNCH investment case and proposal for the Government; and Prof Chigozie Jesse Uneke from Ebonyi State University, the Director of the African Institute for Health Policy and Health Systems in Nigeria. Prof Uneke has published several studies using the EQUIST tool – one on the process of training and another one on the six West African countries' analysis of inequities [27,28]. Alongside the authors of this paper (MC, LB, and HC), Dr Amani Adidja, Deputy Director for Vaccination from the Ministry of Health, Cameroon, also joined remotely as an expert who previously used EQUIST. Dr Adidja developed Cameroon’s ICS for the GFF which influenced the national government.

The group that met at the Summer School in Edinburgh recorded their meeting and all the presentations [29]. They committed to continuing their collaboration, updating and improving the EQUIST, and also applying for grants (see later in the manuscript).

### Introduction of the PATHS tool as a potential add-on to the EQUIST

At the Summer School in Global Health Economics and Priority Setting in Edinburgh, one of the authors of this paper (IR) presented the Pathways to Survival (PATHS) tool, which could become a useful add-on to the EQUIST. It aimed to identify key bottlenecks in health systems’ care provision responsible for most of the severe morbidity and mortality burden. PATHS employs an epidemiological model using decision trees, available evidence, and expert opinion, and also visualises the “architecture” of mortality in the population by following the entire population cohort over a certain period. It explains how initially healthy persons progress through health systems to lethal outcomes at the end of the specified period.

PATHS can expose key bottlenecks in a health system that are the root cause of severe outcomes and/or deaths, the removal of which would have the largest potential to improve those outcomes. It especially brings to attention the causes that are associated with poverty and underprivileged groups. PATHS identifies bottlenecks to implementing interventions and providing care. Weaknesses in health systems where strengthening is most needed will be determined and exposed, and their negative effects on population health quantified. Interventions that can equitably reach vulnerable populations within available resources can be identified and promoted. These results can then be directly translated and communicated to key stakeholders and policymakers, providing data-driven, replicable, and comprehensive policy advice to key stakeholders in which they will be able to take part and ownership themselves, while also engaging local communities. During the Summer School, enthusiasm was expressed toward publishing the PATHS Tool and trying to incorporate it into the EQUIST.

### Progress made in Ghana to allow health planning at the district level

By the end of 2019, a major initiative was undertaken in Ghana related to planning the EQUIST use at the district level, led by Dr Emmanuelle Ankrah Odame, the Director of Policy, Planning, Monitoring and Evaluation (PPME) at the Ministry of Health in Accra. Ghana’s government officials expressed interest in integrating DHIS2 data into EQUIST and taking it down to the level of >200 sub-national administrative districts. Dr Odame led the team from Ghana of several key individuals who worked in software development laboratory in New Delhi, India, and also worked with the University in Oslo. They worked on DHIS2 and ensured that its data was integrated into EQUIST for Ghana, after which they organised several events locally with thematic exercises on, for example, nutrition, immunisation, MNCH, and adolescent issues.

The example from Ghana showed how EQUIST can be used to help district managers determine where they need to act with priority. Prominent officials in the country’s government took EQUIST as a basis for developing their national plans. Two important advances in Ghana were taking the EQUIST to district levels and bringing together district health officers and thematic leads. They all looked at the barriers and bottlenecks and helped move the agenda to the right places using EQUIST. They also tried to bring about a closer linkage between DHIS2 and EQUIST, making decisions based on the data and information that almost reflected the real-time situation, rather than one from several years earlier [30].

## YEARS 2020-2022

During 2020-2022, the coronavirus disease 2019 (COVID-19) pandemic has disrupted many plans, and several of the key figures behind EQUIST’s development and implementation found themselves heavily engaged in addressing the pandemic and its effect in their countries.

### Publication of the PATHS tool

The PATHS Tool was published in 2021 [31]. Using an example of neonatal infections and the potential of early diagnostics, the PATHS tool demonstrated its usefulness in predicting the impact of an emerging intervention or technology on addressing the burden of a major global health problem.

### Considering the integration of the EQUIST with other tools

During the COVID-19 pandemic, further integration of useful priority-setting tools into the EQUIST data science platform was considered. Some tools that have similarities in aims and scope are Davidson Gwatkin’s EquityTool [32] and Health Equity Assessment Tool (HEAT) [33]. Steps 1 and 2 of the EQUIST are also possible through the HEAT.

The INNOV8 guidance tool likewise became available [34]; it bears considerable similarities with the seven steps of implementation of the EQUIST. However, the INNOV8 starts with disease and then looks at its relation to equity in the population, with steps three to seven being largely similar to those of the EQUIST [34], which starts with the population and works from there to determine the most equitable and cost-effective approaches for each disease. As noted, the AHSBA guidance integrates the INNOV8 approach with EQUIST to allow users to identify priority interventions for adolescents’ development.

### Impact of country-level implementation

The most direct impact of EQUIST on reducing maternal and child mortality is achieved through its use for submitting proposals on integrated reproductive, maternal, neonatal, child, and adolescent health and nutrition (RMNCAH&N) outcomes for funding from the GFF and in parallel the World Bank loans from IDA/IBRD. Launched in 2015 by the UN, World Bank, and key partners, the GFF is a key financing platform in support of the UN’s Global Strategy for Women’s, Children’s and Adolescents’ Health as well as the Sustainable Development Goals [22].

To receive funding from the GFF, each applicant country must develop its own national, prioritised plan to improve health outcomes for women, children, and adolescents, known as an ICS, detailing the planned health financing reforms and health system strengthening interventions, as well as the projected impact on health outcomes. EQUIST as a tool has helped governments, institutions, and policymakers in determining priorities and strategies that will form the foundations of the ICS.

The GFF and the agencies that launched it now broadly consider EQUIST as a useful and comprehensive tool available to recipient countries to generate successful ICS. Therefore, potential applicant countries are now advised – and their representatives trained at high-level workshops – to use EQUIST to maximise their chances of being awarded funding. UNICEF, World Bank, and GFF are encouraging national authorities to use EQUIST for developing their prioritized plans for Investment Cases in support of their financing their MNCG portfolios. Indeed, countries that followed this advice were more likely to be successful: the African Institute for Health Policy and Health Systems notes that “investment case studies that were based on EQUIST have so far had a greater success in obtaining GFF funding than if they were not based on EQUIST” [26].

Therefore, the EQUIST has become a useful new paradigm in global health economics, which added the principle of equity to cost-effectiveness. It identified cost-effective and equitable interventions for MNCH and targeted the most effective and equity-focused strategies to fast-track the reduction of maternal and child mortality in LMICs. At least 10 countries have, on the strong recommendation of the funders, used EQUIST to develop their ICSs funding from the GFF for MNCH programmes. In 2017, these investment cases leveraged a combined US$382 million and a further US$290 million as World Bank loans. The GFF rigorously monitors and reports on the impact of the funding leveraged by ICSs in the recipient countries. At the close of 2022, the impacts were most clearly documented and pronounced in Cameroon, Mozambique and the Democratic Republic of Congo, which were the first countries to submit ICSs based on EQUIST. This funding has resulted in improved health outcomes, including the following:

1. In Cameroon, the share of the health budget going to primary and secondary care increased from 8% in 2017 to 21% in 2019. This, coupled with downstream system-strengthening strategies targeted to high-burden regions, resulted in the under-five mortality rate falling from 103 per 1000 live births to 79, and the prevalence of stunting among children under five decreasing from 32% in 2018 to 28.9% in 2019 [30].

2. In Mozambique, the government increased the ratio of health spending to total domestic expenditure, which in the first year of implementation resulted in increases in the number, reach, and capacity of community health workers. These systemic shifts are already improving health outcomes: for example, in 42 prioritised districts, the percentage of births taking place in a healthcare facility jumped from 66% in 2017 to 80% by December 2018. Moreover, nutrition intervention packages were rolled out in the 8 highest-burden regions and provided 3.6 million additional children with a basic nutrition package and services [30].

3. In the Democratic Republic of Congo, the share of the national budget allocated to health rose from 7% in 2016 to 8.5% in 2018. This system-strengthening investment has led to increased access to and the utilisation of key services: between January 2017 and December 2018, 10 000 additional women received antenatal care, 50 000 had assisted deliveries, and 60 000 sought postnatal care [30].

4. In Côte d’Ivoire, where an ICC has been developed [30], the National Coordinator of the Reproductive Health Research Unit confirmed that the use of EQUIST “has improved planning on the choice of strategies and interventions that should have the most impact on maternal, newborn and child deaths in this country. We expect that several thousand lives should be saved as a direct result of these improvements” [26].

In the years ahead, the use of EQUIST will likely be expanded to many more countries and support LMICs to achieve greater advances in their pursuit of achieving Universal Health Coverage for reaching Sustainable Development Goal 3.

### Impact of the EQUIST on national health policies

Besides leveraging funding from the GFF, EQUIST has been used to directly influence national health policy and resource allocation in 26 African countries. In 2018, UNICEF deployed EQUIST in 11 countries in Eastern and Southern Africa to identify bottlenecks and develop prioritised plans to address them. In Angola, these plans contributed to a nearly US$400 million increase in the approved budget for health and education, benefitting more than 16 million children. In West and Central Africa, 15 further countries undertook in-depth equity and bottleneck analyses of health and nutrition programmes using EQUIST and used these to leverage domestic resources [35].

EQUIST was also used by the West African Health Organization in their “Moving maternal, neonatal and child health evidence into policy in West Africa” project, undertaken to promote the implementation of evidence-informed policymaking to improve MNCH in Benin, Burkina Faso, Ghana, Mali, Nigeria, and Senegal. This study concluded: “If the national decision makers are also interested in bridging implementation gaps and in the development of policies that are based on a thorough assessment of how the health system is functioning, particularly with regards to producing equitable health outcomes, then EQUIST is highly imperative” [36].

From 2020, EQUIST has also been used to incorporate Neonatal Tetanus Elimination (NNTE) plans and focused strategies in the NNTE countries. Application programming interphase (API) for automating data entry into EQUIST from DHS and MICS databases, as well as for select indicators from routine data sources such as Health Management Information System (HMIS)/DHIS2 (where stakeholders can confirm the validity and reliability) are being incorporated for increasing number of countries.

Plans for building linkages between AIM/GOAL software and EQUIST for human immunodeficiency virus/acquired immunodeficiency syndrome (HIV/AIDS) programme plans and also for improving adolescent health features in EQUIST are also in progress. The educational activity around EQUIST has served to institutionalise a focus on equity in the planning, prioritisation, and resource allocation for MNCH interventions among decision-makers in LMICs.

### Grant proposal to the National Institute for Health Research, UK: EQUI-RESP-AFRICA

In 2022, the University of Edinburgh, UK and the University of Lagos, Nigeria proposed to the National Institute for Health Research (NIHR) a four-year research programme to implement EQUIST and other tools to improve equity in respiratory disease outcomes in five African countries. The project’s leaders and one of the authors of this paper (IR) (UK) and Obianuju Ozoh (Nigeria) assembled an interdisciplinary group of academics, policymakers, representatives of non-governmental organisations (NGOs), patients, and civil society to address key respiratory diseases in five African countries: Nigeria, Kenya, Cameroon, Cote d’Ivoire, and South Africa. The aim is to build capacity, conduct quality research and an intervention trial, and engage communities and policymakers to achieve demonstrable and equitable reductions in morbidity and mortality from respiratory diseases through evidence-based improvements in prevention, diagnosis, treatment, interventions, and policies.

They intend to conduct summer schools for capacity building in data science and machine learning/artificial intelligence, community engagement, advocacy, and the use of the EQUIST and PATHS. They also intend to link evidence with policy using these two tools to improve equity in outcomes and identify bottlenecks that lead to morbidity and mortality from respiratory causes. A high-level meeting will then be held to prioritise the implementation of equitable interventions that are appropriate to each African context and tailored to patients’ needs. This should allow EQUIST users to incorporate important economic variables and health indicators and broaden EQUIST's scope beyond maternal and child health, addressing non-communicable diseases such as COPD and asthma.

In principle, the EQUIST could be deployed well beyond MNCH issues – not only to non-communicable diseases, but also to global, regional, or national health emergencies, especially where they occur in the context of limited resources. Currently, it is most likely unrealistic that all the required information would be available, so the predictions may be inaccurate. In time, we may collect enough data to make EQUIST’s use more practical even in emergencies.

Efforts to build capacity in EQUIST’s use are still ongoing. It is already incorporated in the master of public health course “Investing in Global Health and Development” at the University of Edinburgh. We also hold Summer Schools in Global Health Economics and Priority Setting through the University of Edinburgh and the International Society of Global Health (ISoGH), where the use of EQUIST is taught and training provided. Also, UNICEF continues to build capacity in its use among LMIC health professionals.

Another important area to consider is collaboration with economists and experts in economic decision-making. This could strengthen EQUIST's methodologies and models to better integrate economic considerations into its analyses, which we plan to do through the EQUI-RESP-AFRICA grant.

## CONCLUSIONS

Since the first evidence-based tools were developed to enable priority setting for health care, health research and support policy in MNCH in Africa [37], this field has received considerable attention. As an example of a novel tool, EQUIST has proven to be valuable in developing national-level health policies and programmes that are both cost-effective and equitable. It was originally developed with the main purpose of addressing the limitations of the traditional CEA in global health, which often overlooked equity considerations or even worsened them.

EQUIST’s primary aim was to create a more effective and efficient health system by explicitly incorporating equity as a key driver in health policy decisions, along with the cost-effectiveness principle. This was in response to the recognition that, while CEA helped reduce mortality rates through interventions like childhood vaccinations, it was insufficient in addressing growing inequalities in health in many low- and middle-income countries (LMICs).

The future of EQUIST includes further integration with other tools and expanding its scope to address broader health issues, including adolescent health and HIV/AIDS, malaria, tuberculosis, and non-communicable causes programme planning. Overall, EQUIST represents a paradigm shift in global health economics, emphasizing the importance of equity alongside cost-effectiveness in health policy decisions. Its development and implementation have had a tangible impact on health outcomes, particularly in LMICs, where EQUIST has been instrumental in reducing maternal and child mortality while addressing health inequities.
